# Early-life exposure to vector-borne pathogens in moose calves from Finland: molecular and phylogenetic evidence

**DOI:** 10.1186/s12917-026-05671-w

**Published:** 2026-06-30

**Authors:** Katarzyna Filip-Hutsch, Antti Oksanen, Petra Heikkinen, Marek Asman, Magdalena Świsłocka-Cutter, Daniel Młocicki, Michał Czopowicz, Krzysztof Anusz, Marja Isomursu, Joanna Werszko

**Affiliations:** 1 Department of Food Hygiene and Public Health Protection, Institute of Veterinary Medicine, Warsaw University of Life Sciences–SGGW, Nowoursynowska 159, Warsaw, 02-776 Poland; 2 Finnish Food Authority, (FINPAR), Elektroniikkatie 3, Oulu, 90590 Finland; 3 Department of Medical and Molecular Biology, Faculty of Medical Sciences in Zabrze, Medical University of Silesia, Katowice, Poland; 4 Department of Zoology and Genetics, Faculty of Biology, University of Białystok, Ciołkowskiego 1J, Białystok, 15-245 Poland; 5 Department of General Biology and Parasitology, Medical University of Warsaw, Chałubińskiego 5, Warsaw, 02-004 Poland; 6 Division of Veterinary Epidemiology and Economics, Institute of Veterinary Medicine, Warsaw University of Life Sciences–SGGW, Nowoursynowska 159C, Warsaw, 02-776 Poland

**Keywords:** *Alces alces*, Hemoparasites, Early-life infection, Northern Europe ecosystems

## Abstract

**Background:**

Vector-borne pathogens are expanding in northern Europe as climate and host–vector communities change, shifting vector distributions, yet baseline data from boreal systems and early-life wildlife hosts remain scarce. Moose (*Alces alces*) represent a useful sentinel species for monitoring pathogen circulation at the wildlife–vector interface because they occupy diverse habitats and are widely distributed across boreal ecosystems. In this study, we investigated the occurrence and phylogenetic location of *Trypanosoma* spp., *Anaplasma phagocytophilum* and *Bartonella* spp. in moose calves during their first season of life, providing insight into early exposure patterns relevant to integrated surveillance. Whole-blood samples from 23 calves (twenty-two hunted animals and one found dead) were screened by PCR and sequenced for 18S rRNA (*Trypanosoma*), 16S rRNA (*A. phagocytophilum*) and rpoB (*Bartonella*).

**Results:**

At least one pathogen was detected in 44% (10/23) of calves. *Trypanosoma* DNA was found in 35% (8/23) and all isolates clustered within the TthII lineage of the *Trypanosoma theileri* complex, including three novel 18S rRNA haplotypes deposited in GenBank. *Anaplasma phagocytophilum* was detected in 17% (4/23), with sequences identical to reference haplotypes reported from Eurasia. *Bartonella* spp. were detected in 9% (2/23), with the rpoB haplotype identical to a lineage previously reported from North America and closely related to ruminant-associated *Bartonella* species.

**Conclusions:**

The detection of multiple vector-borne hemoparasites in moose calves indicates that exposure to diverse pathogens can occur early in life in boreal cervid communities. Phylogenetic relationships suggest that the detected lineages are part of widely distributed pathogen populations rather than locally confined variants. Although this study was not designed to quantify zoonotic risk, documenting pathogen circulation in free-ranging moose may support One Health–oriented surveillance integrating wildlife hosts, vectors, and environmental factors, particularly in regions where hunters and rural communities may be exposed to vector-borne pathogens. These findings establish the grounds for future monitoring efforts aimed at understanding pathogen dynamics, host condition, and emerging vector-borne risks in boreal ecosystems of northern Europe.

## Background

Southwestern Finland represents a distinctive ecological setting characterized by diverse multi-host ungulate communities, fragmented forest–agricultural landscapes, and reported declines in moose calf body mass in northern Europe, potentially associated with environmental changes, population density, and habitat quality [1–3]. These factors may influence pathogen transmission dynamics and host susceptibility. Therefore, molecular surveillance of hemoparasites in moose calves may provide important insights into both wildlife health and broader ecosystem processes. The moose (*Alces alces*), the largest member of the deer family (Cervidae), is widely distributed across northern Eurasia, including Finland, and plays a key role in boreal ecosystems through its influence on vegetation structure and as prey for large carnivores [4]. It is also an important game animal species; e.g., in Finland in 2024, 37,874 moose were hunted [5]. Environmental conditions in boreal regions strongly influence the occurrence and transmission dynamics of vector-borne pathogens. In northern Europe, climate-driven changes in temperature and precipitation may alter vector abundance, seasonal activity, and geographic distribution, thereby increasing opportunities for pathogen transmission in wildlife populations [6]**.** Rising temperatures in northern Europe facilitate the expansion of vectors and the emergence of pathogens previously uncommon in these regions, thereby increasing potential health risks for wildlife, domestic animals, and humans [7, 8].

Moose are exposed to a broad spectrum of vector-borne parasites and therefore constitute a key sentinel species for disease surveillance; since they are the dominant cervid species in Finland. Moose constitute an important sentinel species for vector-borne pathogen surveillance in boreal ecosystems because they use diverse habitats and frequently encounter vector-rich environments across natural and human-modified landscapes [1, 2, 7, 22]. Protozoan parasites of the genus *Trypanosoma* are commonly found in European cervids, including moose [9, 10]. *Trypanosoma theileri* is generally of low pathogenicity and is transmitted primarily by tabanid biting flies [11]. Although infections are usually subclinical, under environmental stress or co-infection they can occasionally cause anemia, fever, and reduced fitness [12].

*Anaplasma phagocytophilum*, a gram-negative bacterium in the order Rickettsiales, causes granulocytic anaplasmosis in humans and animals [13]. In wildlife, it is transmitted by ticks and is frequently detected in moose, red deer (*Cervus elaphus*), roe deer (*Capreolus capreolus*), and fallow deer (*Dama dama*) across Europe [13, 14]. While infections in wild ruminants are often asymptomatic, they may result in weakness, decreased condition, and increased susceptibility to other diseases [15].

*Bartonella* spp. are intracellular bacteria that infect erythrocytes and endothelial cells and have been detected in European wild ruminants, including moose [7, 16]. Infected animals are usually asymptomatic but can serve as reservoirs for transmission to humans and domestic animals. Transmission occurs via ectoparasites such as lice, fleas, ticks, and flies, which can mechanically or biologically spread the bacteria among hosts [17, 18].

Because many vector-borne pathogens circulate among wildlife, domestic animals, and humans, surveillance in free-ranging cervids may contribute to a broader One Health approach integrating animal, environmental, and public health perspectives [17]. Monitoring pathogen circulation in wildlife reservoirs can improve understanding of ecological drivers of disease emergence and support early detection of zoonotic risks associated with environmental perturbations, including climate change, habitat alteration, and shifts in host and vector communities [8, 17].

Understanding pathogen prevalence in moose populations is important for assessing potential risks to livestock and human health and for evaluating how large-scale ecological changes may affect the epidemiology of vector-borne and zoonotic diseases in boreal ecosystems [19].

Despite increasing attention to vector-borne pathogens in European cervids [20, 21], data from boreal archipelago systems remain limited. Moreover, southwestern Finland represents a high-density, multi-host cervid community where ongoing ecological changes, including altered vector dynamics and environmental conditions, may reshape pathogen circulation [22, 23]. Baseline molecular and phylogenetic data from this setting are therefore needed to support surveillance and to inform future studies linking pathogen presence with vector ecology and host condition.

Early-life exposure during the first season of life may be especially informative because calves encounter hematophagous vectors during their first vector-active season while their immune responses are still developing. Age-related differences in infection dynamics have been reported in several wild cervid species, although they remain poorly studied in moose [9]. The aim of this study was therefore to detect and phylogenetically characterize *Trypanosoma* sp., *Anaplasma phagocytophilum* and *Bartonella* sp. in moose calves (*Alces alces*) from southwestern Finland, representing their first season of life. By providing baseline molecular and phylogenetic data for this age group and region, the study contributes to understanding hemoparasite diversity and host–pathogen associations in boreal ecosystems and may support future studies on vector ecology, wildlife health, and zoonotic disease emergence [24].

## Results

### Study population and health status of examined moose

Study population comprised 23 moose calves, 12 males (52%) and 11 females (48%). Slaughter weight was measured in 14 calves and ranged from 34 to 80 kg (median 58 kg). It did not differ significantly between males and females (Mann–Whitney *U* test: *U*_0.05(2),7,7_ = 19.5, *p* = 0.565).

Lesions indicative of pericarditis, including multifocal thickening and proliferation of the epicardium, were found in 16/22 moose calves for which data were available (73%). Pericarditis was not significantly associated with body weight (Mann–Whitney *U* test: *U*_0.05(2),4,9_ = 21.5, *p* = 0.643) or sex of calves (Fisher’s exact test: *p* = 0.635).

**Fig. 1:**
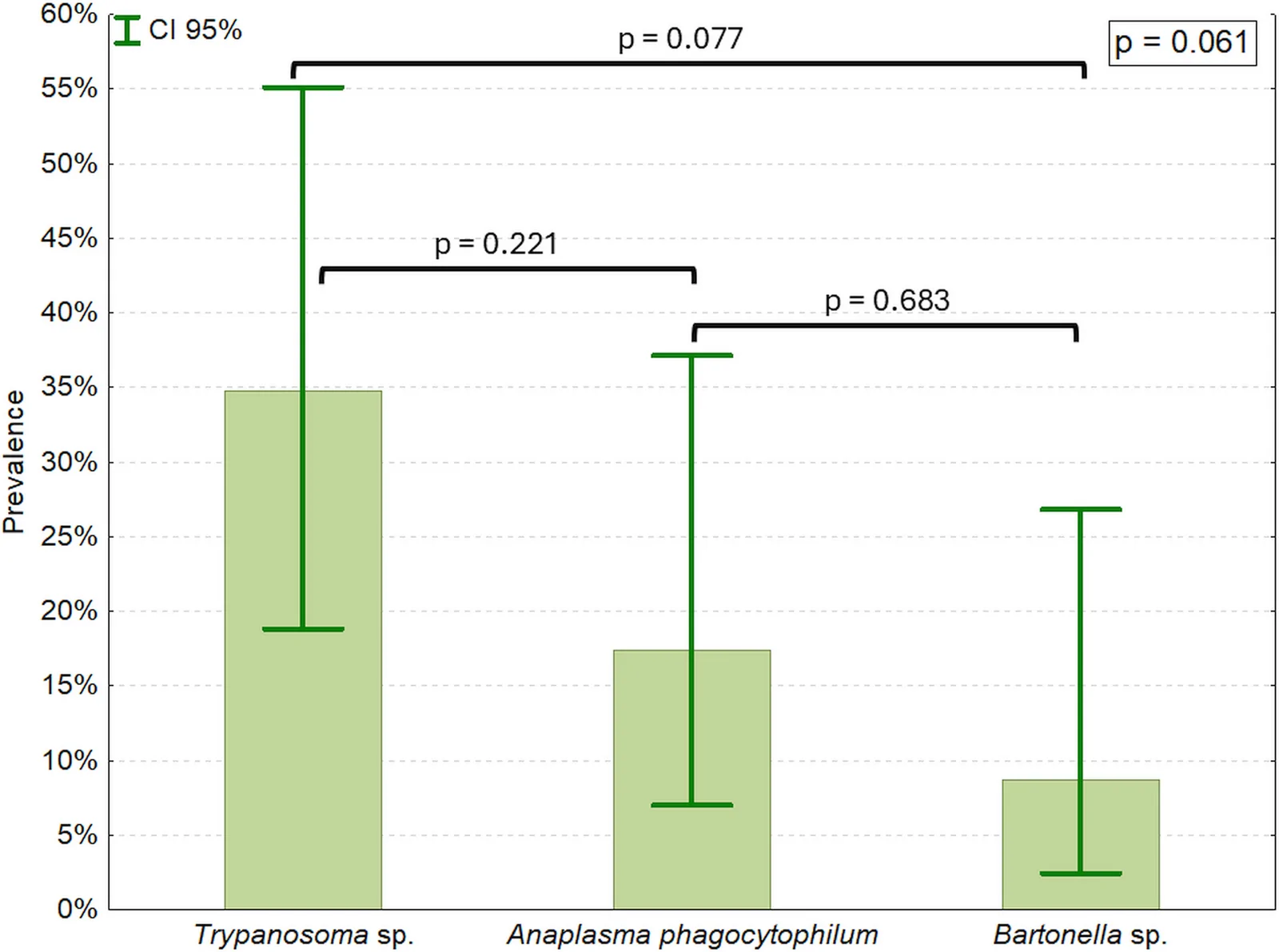
Prevalence of *Trypanosoma* sp., *Anaplasma phagocytophilum* and *Bartonella* sp. in 23 moose examined in the study. Whiskers indicate 95% confidence intervals calculated using Wilson score method. The *p*-value in the upper right corner comes from Cochran’s Q test. The *p*-values above the bars come from McNemar’s test and were not adjusted for multiple comparisons

At least one blood pathogen investigated in this study was detected in 10/23 moose calves (43%; CI 95%: 26%, 63%). The prevalence of infection with individual blood pathogens is presented in Fig. 1.

### *Trypanosoma* sp.

Genetic material of *Trypanosoma* sp. was detected in blood samples of 8/23 moose calves (35%; CI 95%: 19%, 55%). The prevalence of infection was not significantly associated with sex (Fisher’s exact test: *p* = 0.667), body weight (Mann–Whitney *U* test: *U*_0.05(2),5,9_ = 30.0, *p* = 0.350), or the presence of pericarditis (Fisher’s exact test: *p* = 0.136).

**Fig. 2:**
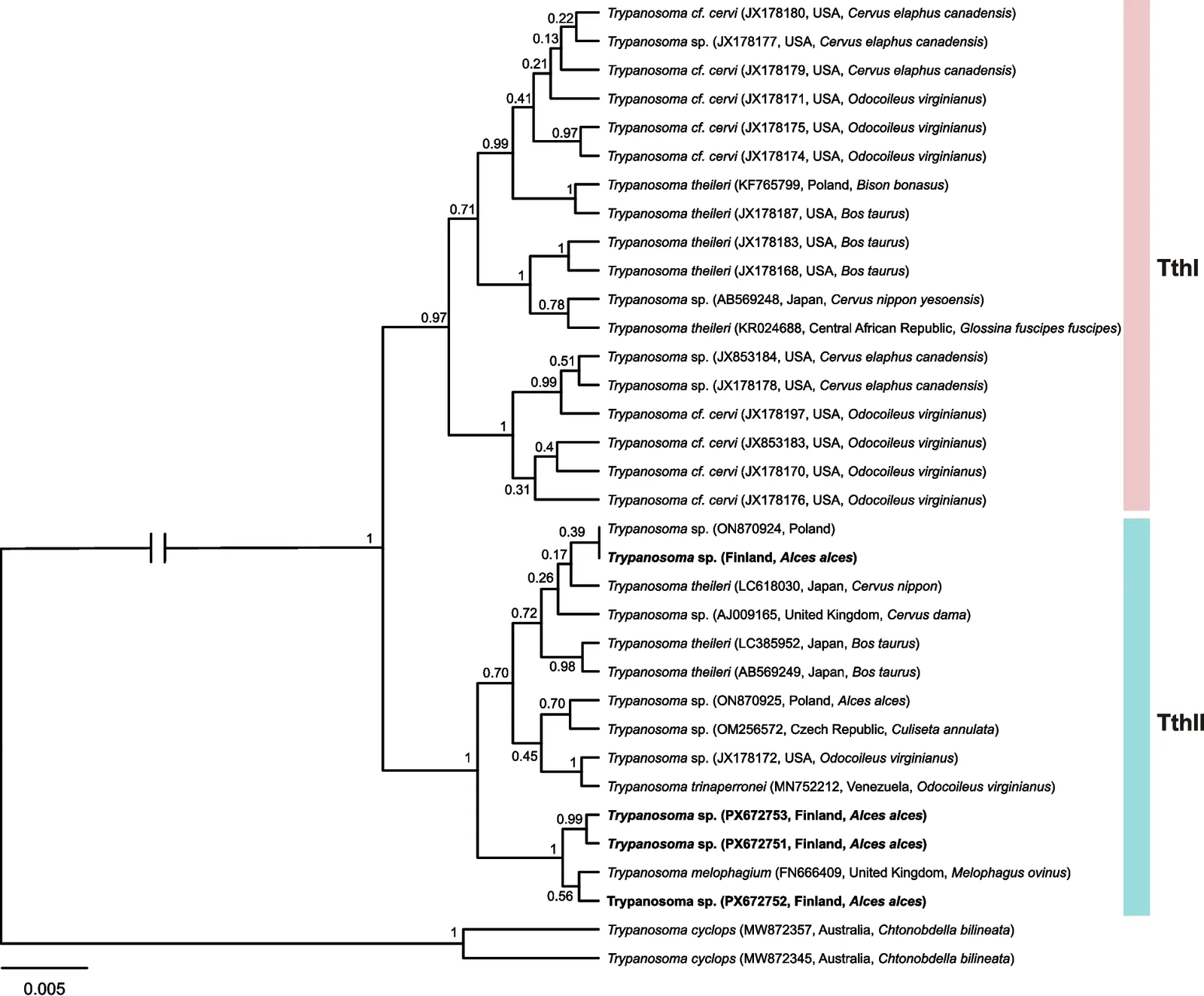
Bayesian tree of *Trypanosoma* sp. haplotypes inferred from 18S rRNA gene fragments. Haplotypes of *Trypanosoma* sp. obtained in this study are marked in bold; numbers following species names represent the accession numbers of sequences downloaded from GenBank. The Bayesian tree was constructed using the GTR + G model of molecular evolution. Values at the nodes indicate posterior probabilities estimated by Bayesian inference using BEAST

The phylogenetic analysis (Fig. 2) based on the 18S rRNA gene sequences revealed that all Finnish *Trypanosoma* isolates clustered within the TthII lineage of the *Trypanosoma theileri* complex. The Finnish isolates PX672751, PX672752 and PX672753 formed a well-supported monophyletic clade together with the sheep parasite *Trypanosoma melophagium* sequences previously reported from the United Kingdom (GenBank accession no. FN666409). Moreover, two other Finnish isolated obtained in the present study were genetically identical to trypanosomes from Poland (GenBank accession no. ON870924) and grouped with additional reference sequences originating from the UK (GenBank accession no. AJ009165, AJ009163) and Japan (GenBank accession no. LC618030).

### Anaplasma phagocytophilum

Genetic material of *A. phagocytophilum* was detected in 4/23 moose calves (17%; CI 95%: 7%, 37%). The prevalence of infection was not significantly associated with sex (Fisher’s exact test: *p* = 0.590), body weight (Mann–Whitney *U* test: *U*_0.05(2),3,11_ = 14.5, *p* = 0.815), or the presence of pericarditis (Fisher’s exact test: *p* = 0.999).

**Fig. 3:**
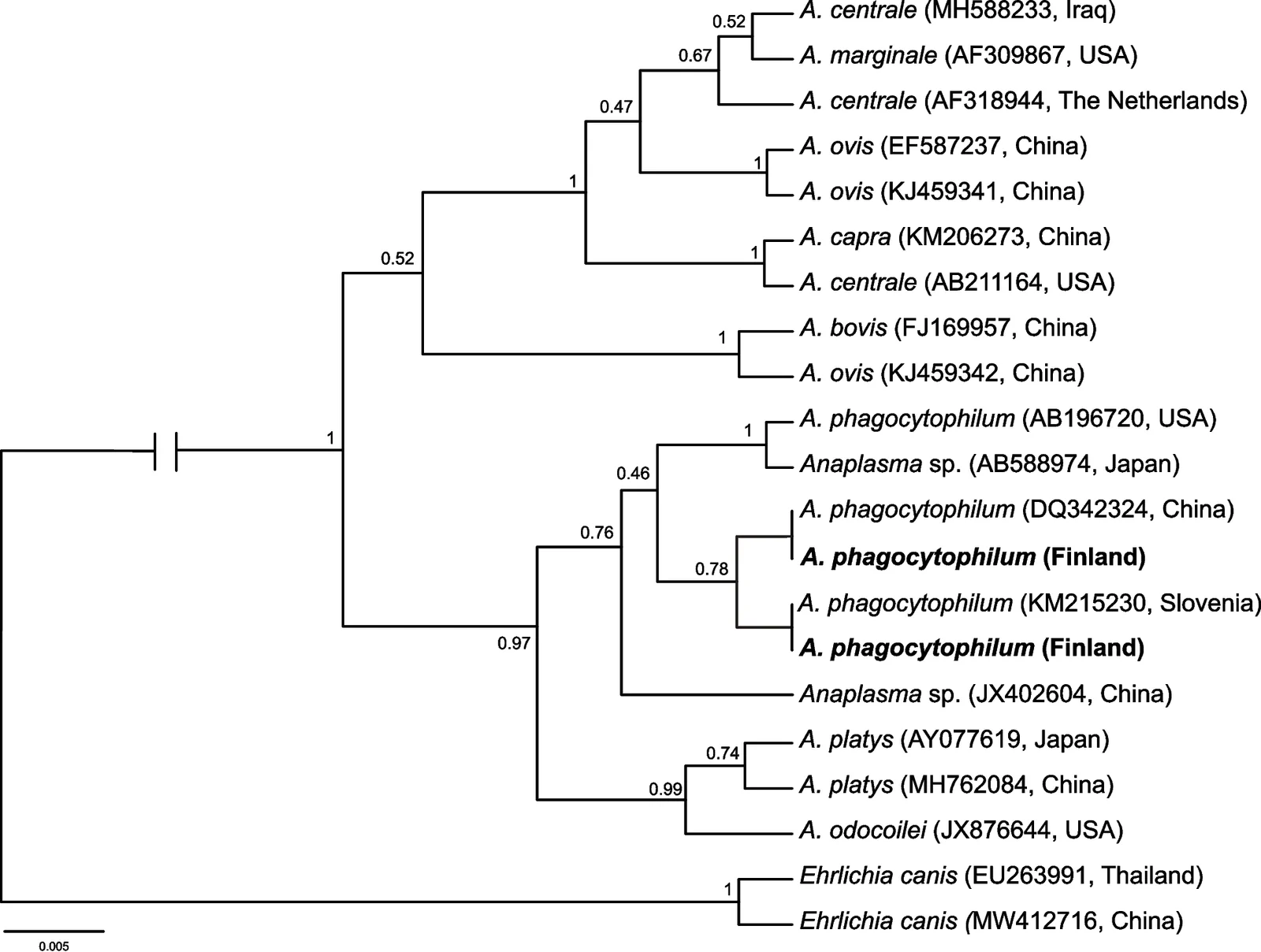
Bayesian tree of *Anaplasma phagocytophilum* haplotypes inferred from 16S rRNA gene fragments. Haplotypes of *A*. *phagocytophilum* obtained in this study are marked in bold; numbers following species names represent the accession numbers of sequences downloaded from GenBank. Multiple isolates sharing identical sequences were represented by a single terminal in the tree. The Bayesian tree was constructed using the HKY + I + G model of molecular evolution. Values at the nodes indicate posterior probabilities estimated by Bayesian inference using BEAST

The phylogenetic tree (Fig. 3) based on 16S rRNA gene sequences revealed that three Finnish *A. phagocytophilum* isolates shared an identical sequence, represented by a single terminal in the tree, which was identical to the *A. phagocytophilum* reference sequence from China (GenBank accession no. DQ343234). One additional isolate was identical to a haplotype of *A. phagocytophilum* from Slovenia (GenBank accession no. KM215230).

### *Bartonella* sp.

Bacteria of the genus *Bartonella* were identified in 2/23 individuals (9%; CI 95%: 2%, 27%). The prevalence of infection was not significantly associated with sex (Fisher’s exact test: *p* = 0.999) or the presence of pericarditis (Fisher’s exact test: *p* = 0.481). For two moose with *Bartonella* sp. infection body weight was unknown.

**Fig. 4:**
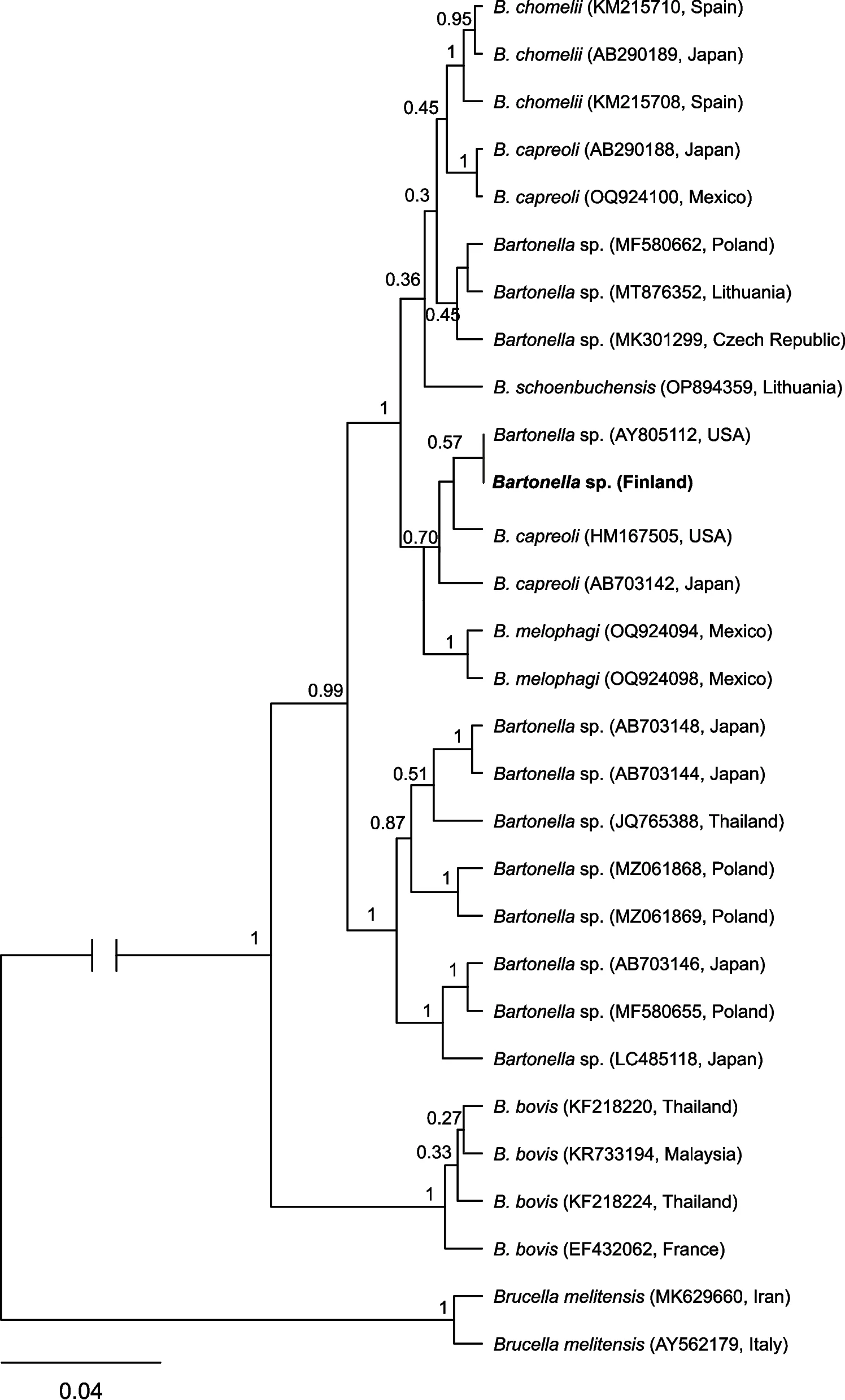
Bayesian tree of *Bartonella* spp. haplotypes inferred from *rpoB* gene fragments. Haplotype of *Bartonella* sp. obtained in this study is marked in bold; numbers following species names represent the accession numbers of sequences downloaded from GenBank. The Bayesian tree was constructed using the GTR + I + G model of molecular evolution. Values at the nodes indicate posterior probabilities estimated by Bayesian inference using BEAST

The phylogenetic analysis of the *rpoB* gene (Fig. 4) showed that the Finnish *Bartonella* sp. isolate is genetically identical to a *Bartonella* sp. sequence from the USA (GenBank accession no. AY805112). This lineage was closely related to *B. capreoli* from the USA (GenBank accession no. HM167505) and Japan (GenBank accession no. AB703142). Those genetics variants of the *rpoB* gene clustered together also with sequences of *B. melophagi* from Mexico (GenBank accession no. OQ924094 and OQ924098).

### Co-infections

Co-infections were observed in 4/23 moose calves (17%): three involved *Trypanosoma* sp. and *A. phagocytophilum*, and one involved *Trypanosoma* sp. and *Bartonella* sp.

No significant difference in prevalence between three examined pathogens was observed (Cochran’s Q test: Q_0.05(2),2_ = 5.60, *p* = 0.061; Fig. 1). However, as the *p*-value of omnibus Cochran’s Q test was very close to statistical significance, pairwise comparisons were performed. None of prevalences turned out to differ significantly from the others with the difference between *Trypanosoma* sp. and *Bartonella* sp. being closest to statistical significance (McNemar’s test: χ^2^_0.05(2),1_ = 3.125, *p* = 0.077).

## Discussion

Among 23 moose examined in the present study, 10 individuals (44%) were infected with at least one blood parasite, indicating that exposure to vector-borne pathogens may occur commonly during the first season of life. Although direct comparison with previous studies is difficult because of differences in host age, sampling design, geographic region, and targeted pathogens, the detected prevalence values fall within the range previously reported for hemoparasites in European cervids and moose populations [10, 25–27]. PCR screening confirmed the presence of three targeted groups of hemoparasites — *Trypanosoma* sp., *A. phagocytophilum*, and *Bartonella* sp., confirming that these microorganisms are established components of the pathogen communities in moose in northern Europe [13, 28]. Unlike previous studies primarily focused on adult cervids, this work provides age-specific molecular and phylogenetic data from moose calves, representing early-life exposure during the first vector season.

Phylogenetic analyses of *A. phagocytophilum*, *Bartonella* sp., and *Trypanosoma* sp. isolates revealed clear evolutionary relationships between Finnish lineages and those found in Eurasia. The clustering patterns of all three examined pathogens show similarities to previously published isolates originating from various ruminant host species and their associated vectors. The trypanosome haplotypes clustered within the TthII lineage of the *T. theileri* complex, which is characteristic of both wild and domestic ruminants in Europe [10, 29]. This confirms that these trypanosomes belong to a broadly distributed and genetically cohesive group of Megatrypanum parasites. The clustering of Finnish isolates with *T. melophagium* previously reported from the United Kingdom supports the notion that TthII encompasses *T. melophagium -*like genotypes transmitted primarily by sheep keds or related vectors. Previous molecular analyses revealed that *T. melophagium* sequences are highly similar to those of *T. theileri*, suggesting that *T. melophagium* represents a lineage of the *T. theileri* complex that has adapted to specific hosts and vectors [30]. The genetic identity of two other Finnish isolates with Polish *Trypanosoma* sequences, and their grouping with reference sequences from both the UK and Japan, indicates that major sublineages within TthII are not restricted to a single geographic region but are shared across Europe and Asia [31]. However, the observed similarity between the Finnish isolates and sequences from geographically distant regions should be interpreted within a broader methodological context. In particular, the high degree of conservation of the 18S rRNA gene among *T. theileri* lineages often results in limited sequence divergence even across the wide range of hosts and vectors. This highlights the need for multilocus and genomic analyses to more accurately define phylogenetic relationships and to discriminate host-adapted variants within this complex [32].

The phylogenetic position of the Finnish *Bartonella* sp. isolate within a cluster identical to haplotypes previously reported from the United States of America confirms a close molecular relationship across large geographic distance. This finding is consistent with previous studies documenting widespread distribution of *Bartonella* strains among wild and domestic mammals [30]. Furthermore, the haplotype identified in the present study exhibited high similarity to *B. capreoli* and *B. melophagi*—species primarily associated with domestic ruminants but also reported in cervid hosts [33], suggesting potential host overlap and circulation across diverse mammalian reservoirs.

The phylogenetic relationships identified in this study indicate that Finnish *A. phagocytophilum* isolates cluster with strains previously reported from China and Slovenia, suggesting the circulation of closely related lineages across Eurasia that are associated with a broad range of hosts and vectors [34, 35]. This is consistent with the fact that several blood-borne parasites of wild ungulates exhibit broad host associations and highly adaptive survival strategies—features that may be reflected in our phylogenetic patterns [13]. *Anaplasma phagocytophilum* and *Bartonella* spp. in particular are known to infect multiple ruminant hosts and display high genetic heterogeneity, while certain trypanosomes of cervids can also circulate within closely related host taxa [28, 34, 36–38].

All examined pathogens are capable of persisting within their hosts for extended periods—often spanning the interval between successive vector seasons—which facilitates long-term maintenance of infection hotspots in wild populations [36, 39, 40]. Consequently, the presence of co-occurrence of competent hosts (e.g., cervids) together with appropriate vectors (ticks for *A. phagocytophilum*; deer keds (*Lipoptena cervi*) and other biting flies for *Bartonella* spp.; biting flies, including keds, for trypanosomes) plays a critical role in sustaining transmission cycles in natural ecosystems [27, 28, 40, 41].

Ecological factors, including vector and host density, habitat structure, and climatic conditions, are likely to influence pathogen occurrence in moose. Consequently, observed spatial patterns may reflect regional variations in vector distribution and abundance, as documented for tick-borne and fly-borne pathogens across Europe [35, 42–44].

Overall, the pathogens detected in this study are considered as having rather low pathogenicity in wild ruminants. *Trypanosoma* and *Bartonella* are typically considered as low-pathogenic or non-pathogenic; however, their epidemiology and potential subclinical effects on host health and fitness remain poorly understood [10, 29, 45]. Infections with *A. phagocytophilum* are usually subclinical; nevertheless, disease have been reported in association with specific genetic variants of the bacterium, particularly in young animals such as calves, which may be more susceptible to infection and disease development [36, 39]. The potential causal relationship between the blood parasites detected in this study and the poor growth performance and low carcass weights observed in moose calves in the southwestern Finnish archipelago cannot be evaluated, as comparative data from areas with higher calf growth rates are currently lacking. Additionally, statistical analysis did not reveal any associations between the presence of blood pathogens and pericarditis in the examined moose calves.

The presence of *A. phagocytophilum* and ruminant-associated *Bartonella* lineages in moose calves may have One Health relevance. In areas with abundant cervids and shared vectors, wildlife can contribute to pathogen maintenance and spillover risk at the wildlife–livestock–human interface. Both genera include zoonotic agents transmitted by arthropods that may also bite humans. *A. phagocytophilum*, the agent of human granulocytic anaplasmosis, is linked to Ixodes ticks and wildlife hosts, with human cases associated with specific zoonotic subclusters [34, 46]. In North America, reported human anaplasmosis has increased over the last two decades, highlighting the public-health importance of monitoring enzootic cycles (CDC Epidemiology and Statistics 2024). Several *Bartonella* species are zoonotic, and vectors such as deer keds can occasionally bite humans, causing conditions like deer ked dermatitis [17, 47]. While our study did not quantify zoonotic risk, documenting pathogen circulation in a dominant wild host provides ecological context relevant to integrated wildlife–vector surveillanceand supports integrated wildlife–vector–environment surveillance.

## Conclusion

Despite limitations related to sample size, geographic scope, and sampling period, the present study provides valuable baseline data and identifies key directions for future research, including larger-scale, longitudinal investigations and integrated host–vector approaches. The prevalence values reported here should be interpreted as indicators of exposure rather than population-level estimates, given the age-specific and limited sampling design. Sampling was conducted during a limited seasonal window and therefore may not reflect annual variation in pathogen exposure. Vector activity in northern Europe is strongly seasonal, with tick, deer ked, and biting fly abundance typically increasing during warmer months, which may influence both transmission intensity and detectability of infections. Consequently, prevalence patterns observed in calves during their first vector-active season may differ from those observed later in summer, autumn, or in older age classes. In addition, most samples originated from hunter-harvested animals, which may not fully represent the broader moose population. Opportunistic sampling can introduce bias related to animal condition, behavior, habitat accessibility, or hunting pressure, potentially affecting pathogen detection patterns. The restricted geographic scope further limits extrapolation of the findings to other boreal regions with different host densities, habitat composition, vector communities, or climatic conditions.

The detection of *Trypanosoma* sp., *A. phagocytophilum*, or *Bartonella* sp. in over 40% of the examined moose calves demonstrates that exposure to vector-borne hemoparasites can occur early in life in southwestern Finland. Although infections in wild ruminants are typically considered subclinical, calves may represent an age class with distinct infection dynamics and early exposure to vectors, and persistent or multiple infections could plausibly contribute to reduced performance under nutritional or environmental constraints [7, 13, 36]. In this context, the documented decline in calf body weights in the region and the multiple cases of pericarditis underscore the need to evaluate whether chronic hemoparasite exposure may act as an additional stressor alongside density-dependent effects, habitat quality, and climatic variability.

From an eco-epidemiological perspective, phylogenetic analyses indicate that the detected lineages are part of widely distributed pathogen populations rather than strictly local variants. The clustering of Finnish sequences with reference haplotypes from distant regions is consistent with broad host and vector associations and, in some cases, reflects the limited resolution of conservative molecular markers [13, 34]. Accordingly, future studies should combine broader spatial and temporal sampling with higher-resolution typing methods and vector screening to better resolve transmission pathways and host–vector specificity in northern cervid communities.

Age-targeted molecular surveillance may complement traditional wildlife monitoring by capturing early infection events and providing insight into pathogen establishment at the population level.

In conclusion, moose calves in Finland harbour multiple vector-borne hemoparasites with relevance for wildlife health, livestock interfaces, and One Health–oriented surveillance. Establishing robust baselines and tracking temporal changes through long-term monitoring will be essential as vector distributions continue to shift under climate change [6, 8]. Integrating pathogen detection with vector data, host condition metrics, and co-infection analyses may allow boreal cervid systems to contribute to early detection of changing vector-borne pathogen dynamics for emerging vector-borne risks in northern Europe.

## Methods

### Study area and population

**Fig. 5:**
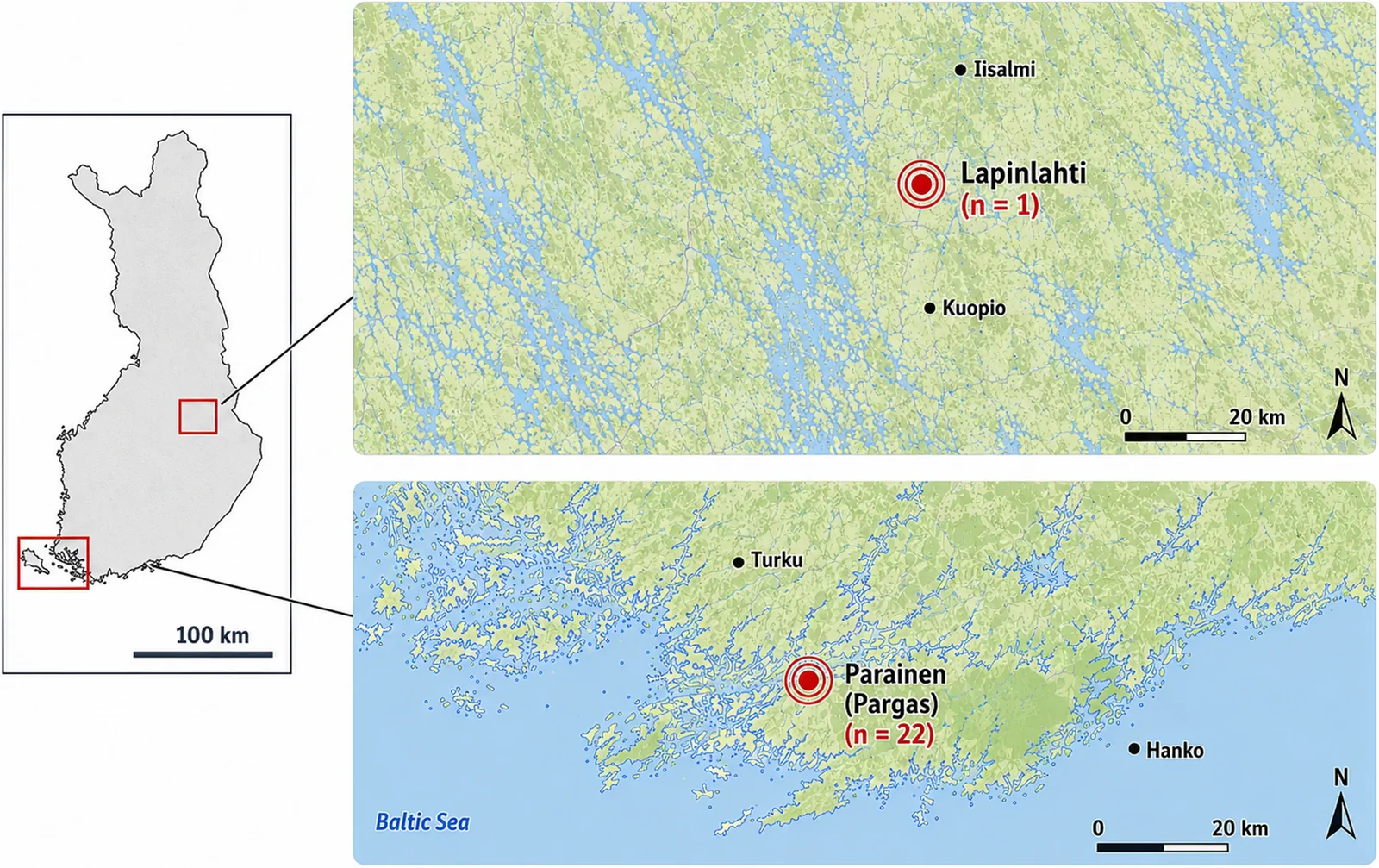
Study area and sampling location

The study was conducted primarily in Parainen (Pargas), Southwest Finland (Fig. 5), an archipelago area situated in the hemiboreal zone with temperate climate, annual mean temperature varying between + 6 and + 7 °C. The area is densely inhabited by wild cervids. The moose density in the region was among the highest in Finland in 2022 (ca. 4 individuals/10 km^2^ post-harvest), and the white-tailed deer *Odocoileus virginianus* was highly abundant (> 30 individuals/10 km^2^) [48]. According to annual snow track counts, the density of the roe deer *Capreolus capreolus* in southwestern Finland has been among the highest in Finland [49]. In Parainen and in the whole southwestern Finland, declining moose calf body weight phenomenon, indicating slow growth, has been observed during this millennium [3].

### Samples

In October 2022, samples were obtained from 22 moose calves (approximately 6 months old) hunted by members of seven hunting clubs in Parainen, Finland. In addition, one sample originated from a small, emaciated moose calf found dead in October 2022 in eastern Finland (Lapinlahti).

Data on age, sex, body weight, and anatomopathological lesions were recorded by qualified hunters. Blood samples were collected post-mortem into EDTA tubes and transported to the laboratory at + 4 °C.

Approval from an ethics committee was not required, as no live animals were used in this study, and none were killed specifically for research purposes.

### Molecular analysis

DNA was extracted from 100 µL of blood at the Finnish Food Authority in Oulu using an QIAamp DNA Mini Kit (Qiagen, Hilden, Germany) (manufacturer’s protocol). The extracted DNA was transported to the Warsaw University of Life Sciences and stored at − 18 °C until molecular analysis.

To minimize contamination risk, pre- and post-PCR procedures were performed in separated areas, and nuclease-free water as negative control were included in each PCR run. The selected genetic markers were chosen based on their widespread use and proven resolution in molecular epidemiological studies of hemoparasites.

Trypanosomes were detected using nested PCR following the protocol described by Noyes et al. [50]. Two pairs of primers were employed: TRY927F (5′-CAGAAACGAAACACGGGAG-3′) and TRY927R (5′-CCTACTGGGCAGCTTGGA-3′), as well as SSU561F (5′-TGGGATAACAAAGGAGCA-3′) and SSU561R (5′-CTGAGACTGTAACCTCAAAGC-3′), which amplify an approximately 560bp fragment of the 18S rRNA gene.

In turn, *A. phagocytophilum* was detected by the nested PCR, and a two pair of primers, ge3a (5’ CACATGCAAGTCGAACGGATTATTC 3’) and ge10r (5’ TTCCGTTAA-GAAGGATCTAATCTCC 3’), and ge9f (5’AACGGATTATTCTTTATAGCTTGCT 3’) and ge2 (5’ GGCAGTATTAAAAGCAGCTCCAGG 3’), specific to the 16S rRNA gene as proposed by Massung et al. [51]. The first pair of primers amplified a 932 bp fragment of 16S rRNA gene. In turn, the pair of inner primers amplified a 546 bp fragment of this gene.

The molecular detection of *Bartonella* sp. was performed using nested PCR according to Renesto et al. [52]. The amplification employed primers 1400Fa (5′-CGCATTGGCTTACTTCGTATG-3′) and 2300R (5′-GTAGACTGATTAGAACGCTG-3′), which target an approximately 825 bp fragment of the *rpoB* gene.

PCR products were visualized on a 1%—2% agarose gel stained with ethidium bromide using a ChemiDoc MP imaging system (Bio-Rad, Hercules, USA) or a Vilber Lourmat device (Vilber Lourmat, Collegien, France). The resulting products were compared to the Nova 100 bp DNA Ladder (Novazym, Poznań, Poland) or PerfectTM 100-1000bp DNA Ladder (EURx, Gdańsk, Poland). PCR products were purified using the QIAEX II Gel Extraction Kit (Qiagen, Hilden, Germany) or GeneMATRIX Agarose-OUT DNA Purification Kit (EURx, Gdansk, Poland) according to the manufacturer’s protocol and then sequenced by Genomed (Warsaw, Poland).

The purified sequences were assembled into contigs using ContigExpress, Vector NTI Advance 11.0 (Invitrogen Life Technologies, New York, USA) [53]. Sequence identities were determined using BLAST (Basic Local Alignment Search Tool) [54] against reference sequences available in GenBank.

### Statistical and phylogenetic analyses

Nuclear *Trypanosoma* sp. 18S rRNA gene fragments, *A. phagocytophilum* 16S rRNA gene fragments and *Bartonella* sp. RNA polymerase beta-subunit (*rpoB*) gene fragments and were manually edited and aligned using BioEdit v.7.0.5.3 [55]. All insertion–deletion sites were re-examined against the corresponding chromatogram traces to identify and correct any discrepancies resulting from automated base calling. Three 18S rRNA haplotypes generated in the present study and representing novel *Trypanosoma* sp. haplotypes were submitted to GenBank (accession numbers: PX672751 – H1, PX672752 – H2, PX672753 – H3).

To infer evolutionary relationships between the *Trypanosoma* sp., *A*. *phagocytophilum*, and *Bartonella* spp. haplotypes detected in our study and representative sequences retrieved from GenBank, we constructed phylogenetic trees based on the 18S rRNA (for *Trypanosoma* sp.), 16S rRNA (for *A*. *phagocytophilum*), and *rpoB* (for *Bartonella* sp.) markers. Taxonomic assignment of all reference sequences was verified using the metadata available in the NCBI database.

Model selection based on the Akaike Information Criterion implemented in jModelTest v.0.1.1 [56], identified GTR + G as the optimal substitution model for 18S rRNA, HKY + I + G for 16S rRNA, and GTR + I + G for the *rpoB* gene.

Bayesian phylogenetic inference for all three nuclear markers was performed in BEAST v.1.7.2 [57], applying the Yule speciation process as the tree prior [58]. The Markov chain Monte Carlo (MCMC) analysis consisted of a single run of 30 × 10⁶ iterations, with sampling every 3000 generations. The first 10% of sampled states were discarded as burn-in. Convergence and the effective sample sizes (ESS > 200 for all parameters) were assessed in Tracer v.1.6 [57] to ensure adequate mixing of the MCMC chains. Posterior tree distributions were summarized using TreeAnnotator v.1.7.2 [57] and visualized with FigTree v.1.3.1 [59]. Outgroup taxa were selected from publicly available sequences appropriate for each genetic marker. Pairwise *p*-distances were calculated in MEGA v.11 [60] using the pairwise deletion option.

Slaughter weight was described as median and range, and compared between unrelated groups using the Mann–Whitney *U* test. Categorical variables (sex, pericarditis, infections) were summarized as counts and proportions, and compared between unrelated groups (infected vs uninfected with each pathogen separately) using Fisher’s exact test. Prevalences of three infections among 23 moose calves were compared first using Cochran’s Q test as an omnibus test and, if significant, pairwise comparisons were performed using McNemar’s test with Yates correction for continuity. The 95% confidence interval (CI 95%) for proportions was calculated with Wilson’s score method [61]. All *p*-values were two-sided and considered to be statistically significant at the 0.05 level. No correction for multiple comparisons was used not to reduce the power of pairwise comparisons. Statistical analysis was carried out in TIBCO Statistica 13.3 (TIBCO Software Inc., Palo Alto, CA) [62].

## Data Availability

The nucleotide sequences generated in this study have been deposited in GenBank under accession numbers PX672751—PX672753. Other datasets generated and/or analysed during the current study are available from the corresponding author on reasonable request.
